# Hindering the illegal trade in dog and cat furs through a DNA-based protocol for species identification

**DOI:** 10.7717/peerj.4902

**Published:** 2018-06-05

**Authors:** Luisa Garofalo, Alessia Mariacher, Rita Fanelli, Rosario Fico, Rita Lorenzini

**Affiliations:** 1 Istituto Zooprofilattico Sperimentale delle Regioni Lazio e Toscana “M. Aleandri”, Centro di Referenza Nazionale per la Medicina Forense Veterinaria, Rieti, Italy; 2 Istituto Zooprofilattico Sperimentale delle Regioni Lazio e Toscana “M. Aleandri”, Centro di Referenza Nazionale per la Medicina Forense Veterinaria, Grosseto, Italy

**Keywords:** DNA testing, Cytochrome b, EU Regulation 1523/2007, Control Region, Mitochondrial ND1, Fur industry

## Abstract

In Western countries dogs and cats are the most popular pets, and people are increasingly opposed to their rearing for the fur industry. In 2007, a Regulation of the European Union (EU) banned the use and trade of dog and cat furs, but an official analytical protocol to identify them as source species was not provided, and violations of law are still frequent in all Member States. In this paper we report on the development and validation of a simple and affordable DNA method for species detection in furs to use as an effective tool to combat illegal trade in fur products. A set of mitochondrial primers was designed for amplification of partial cytochrome b, control region and ND1 gene in highly degraded samples, like furs and pelts. Our amplification workflow involved the use of a non-specific primer pair to perform a first test to identify the species through sequencing, then the application of species-specific primer pairs to use in singleplex end-point PCRs as confirmation tests. The advantage of this two-step procedure is twofold: on the one hand it minimises the possibility of negative test results from degraded samples, since failure of amplification with a first set of primers can be offset by successful amplification of the second, and on the other it adds confidence and reliability to final authentication of species. All designed primers were validated on a reference collection of tissue samples, obtaining solid results in terms of specificity, sensitivity, repeatability and reproducibility. Application of the protocol on real caseworks from seized furs yielded successful results also from old and dyed furs, suggesting that age and chemical staining do not necessarily affect positive amplifications. Major pros of this approach are: (1) sensitive and informative primer sets for detection of species; (2) short PCR amplicons for the analysis of poor quality DNA; (3) binding primers that avoid contamination from human DNA; (4) user-friendly protocol for any laboratory equipped for analysis of low-copy-number DNA. Our molecular procedure proved to be a good starting point for enforcing the EU Regulation against dog and cat fur trade in forensic contexts where source attribution is essential to the assignment of responsibilities.

## Introduction

About 55 million animals are assumed to die every year for their fur (Kerswell, 2009). China is the world’s largest supplier and exporter of fur products (garments, clothing accessories, but also children’s soft-toys), while the European Union (EU) represents the major importer. Nonetheless, the feeling of outrage against animal furs is currently widespread in Europe, North America and Asia, where anti-fur campaigns also contribute to raise public awareness through the support of many popular figures (People for the Ethical Treatment of Animals, PETA, http://www.peta.org).

In Western countries, dogs and cats are the most popular and beloved companion pets. Consequently, people find it unacceptable to farm these animals for their furs, nor do they want to inadvertently buy products containing such fur. On the contrary, dog and cat fur trade is a thriving market in many Asian countries, China in particular, where the rearing of these domestic species for fur-pelt industry is practiced legally, and where laws on animal welfare are lacking. The United States first prohibited dog and cat furs trade in 2000 (Government of the United States, 2000), as did Australia in 2005 (Government of Australia, 2005). Switzerland banned the import of these items in 2006, but killing cats to make furs at home is a lawful practice (Paterson, 2008; PETA, 2008). Italy adopted a legislation against the production and marketing of fur from dogs and cats on its own territory in 2004 (Italian Parliament, 2004). Conversely, it still remains legal to import dog and cat furs into Canada (Government of Canada, 2010) where, however, these products are often deliberately mislabelled in order not to hurt the sensitivity of potential buyers. Following the autonomous initiative of some European countries, the EU officially banned the import to or the export from all Member States of dog (*Canis lupus familiaris*) and cat (both domestic and non-domestic *Felis silvestris*) furs, and all products containing fur from these animals, with a Regulation (n.1523) dated 2007 (European Parliament, 2007). The Regulation allowed that the rules on type and severity of penalties to infringements of the provisions were laid down under each national law.

One of the first steps to ensure that the ban is complied with and the violations are found out is the ability to discriminate dog and cat furs from products that are made with legal fur-bearing species. The EU Regulation states that microscopy (morphological analysis of hairs), molecular testing (PCR-based DNA analysis) and MALDI-TOF mass spectrometry (chemical analysis of hair keratin peptides) can be used to identify dog and cat furs. Each Member State is left free to adopt one of such techniques.

Microscopical identification of mammalian hairs requires extensive training and experience of the examiner, application of multiple analytical methods (cf. Chernova, 2003), and a collection of reference specimens for comparison with questioned samples (Tridico et al., 2014). MALDI-TOF technology has been rarely used to identify chemically processed hairs from animal fur samples, and very few studies have been published so far (Hollemeyer, Altmeyer & Heinzle, 2007; Hollemeyer et al., 2012). The equipment is also economically demanding. Both methods, however, identify hairs with a high degree of confidence only at the family level, in few cases down to the species level, but never in the case of closely related species of carnivores (Hollemeyer et al., 2012; Tridico et al., 2014). Conversely, DNA-based technology overall has a higher diagnostic power, being at the same time a toolkit close-at-hand of every laboratory with basic molecular equipment.

Mitochondrial DNA (mtDNA) is commonly used to identify mammal species (Hsieth et al., 2001; Hebert, Ratnasingham & deWaard, 2003; Dawnay et al., 2007; Ferri et al., 2009; Johnson, Wilson-Wilde & Linacre, 2014 for a review) due to its inherent features, e.g. interspecific variability and basically lack of recombination (which reduces intraspecific diversity). Sequence variation at mtDNA coding genes, like the most widely used cytochrome b (cytb) and cytochrome oxidase I (COI), however, may fail to distinguish closely related species. Furthermore, the COI barcoding marker was revealed to be unsuitable when applied to certain mammal species (Tobe, Kitchener & Linacre, 2009). In such cases, the use of a second, more variable mtDNA sequence, like the non-coding displacement loop (D-loop or control region), can help disentangling species similarity (cf. Gupta, Thangaraj & Singh, 2011; Iyengar, 2014 and references herein). An advantage in using mtDNA is the presence of a high copy number of mitochondrial molecules per cell that allows recovery of DNA from traces and degraded biological sources, such as forensic evidence (e.g. old bones, blood stains; Alacs et al., 2010; Linacre & Tobe, 2011; Iyengar, 2014), noninvasive samples (e.g. hairs, faeces; Waits & Paetkau, 2005) and animal furs and pelts (Sahajpal et al., 2009; Jun et al., 2011; Pilli et al., 2014; this study). Typically, species are identified using universal mitochondrial primers (Kocher et al., 1989; Irwin, Kocher & Wilson, 1991), which can also amplify non-target contaminant species. In fur samples, the main source of contamination is human DNA, due to manufacturing and handling. Universal primers are also prone to (co)amplification of nuclear numts (Lopez, Cevario & O’Brien, 1996; Song et al., 2008) that mislead and can yield incorrect results. With the main purpose of complying with the EU Regulation, we developed and validated a simple and affordable DNA-based method that overcomes some of these drawbacks, through a combined use of newly-designed non-specific and specific mitochondrial primers for species authentication in furs. Reliability of the protocol was finally tested on unknown evidence samples of furs/hides from real caseworks.

## Materials and Methods

### Reference samples

Tissues from authenticated specimens of Canidae (10 dogs *Canis lupus familiaris*, five wolves *C. lupus*, two coyotes *C. latrans*, two jackals *C. aureus*, one raccoon dog *Nyctereutes procyonoides*, 10 red foxes *Vulpes vulpes*) and Felidae (eight domestic cats *Felis silvestris catus*, two wild cats *Felis silvestris silvestris*, three Eurasian lynxes *Lynx lynx* and one tiger *Panthera tigris*) were used as reference samples to develop and validate our tests. Additional non-canid/felid species of Mustelidae (two beech martens *Martes foina*, one otter *Lutra lutra*), Leporidae (two rabbits *Oryctolagus cuniculus*, two brown hares *Lepus europaeus*), Sciuridae (two red squirrels *Sciurus vulgaris*) and Procyonidae (one raccoon *Procyon lotor*), that are commonly used in furriery, were also analysed to evaluate specificity of the tests. Species identity of the reference specimens was assigned by expert veterinarians during necropsy, through morphological examination of animals accidentally died, legally hunted or poached. Tissues of *C. latrans* and *C. aureus* were provided by zoologists. In no case were animals sacrificed for the purpose of the present study. All procedures for the handling of specimens and samples followed the criteria laid out by the Istituto Zooprofilattico Sperimentale del Lazio e della Toscana. Three individuals of *Homo sapiens* (sampled by buccal swabs) were also included among the reference species to monitor cross-amplification.

### Questioned samples

**Figure 1:**
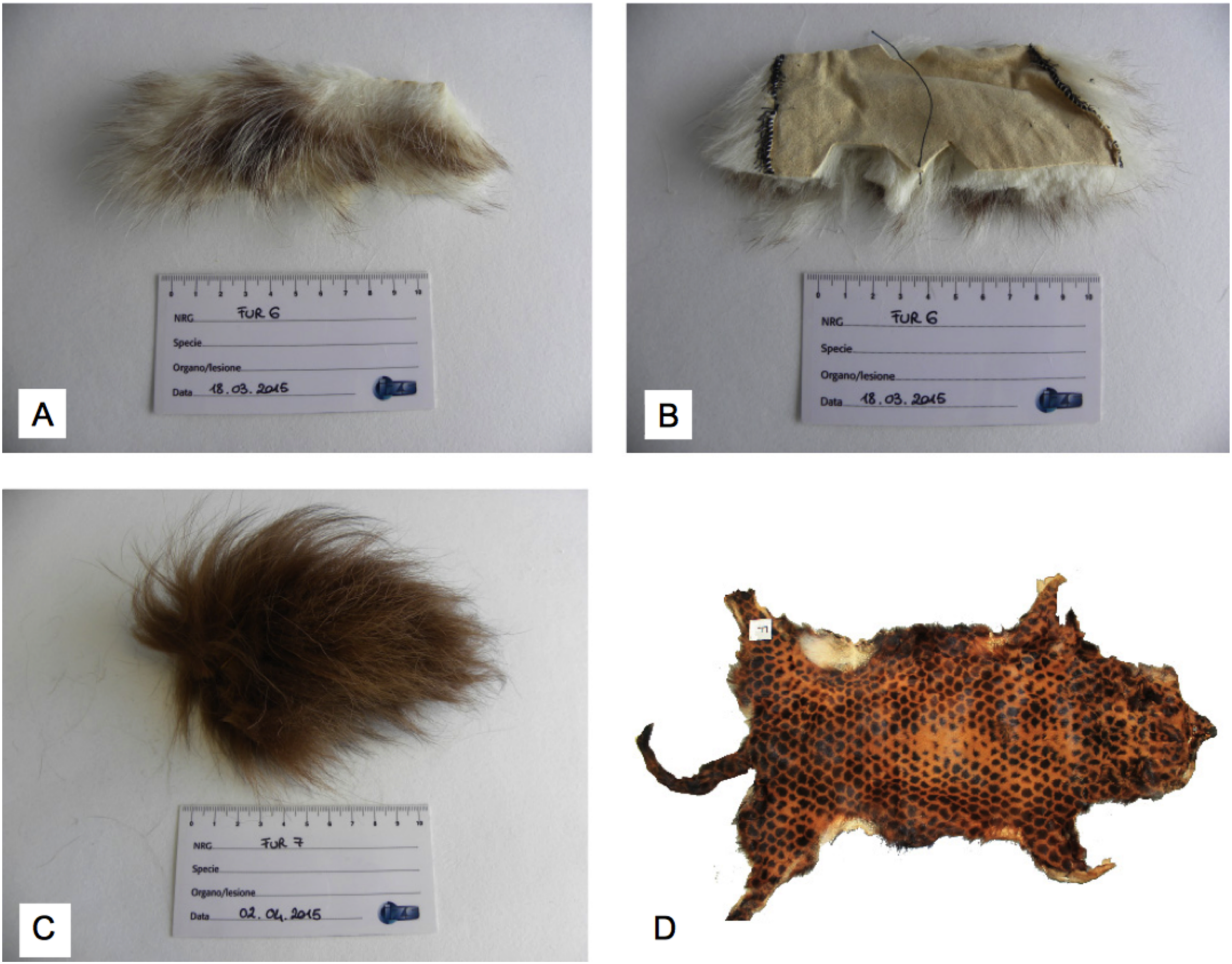
Examples of casework samples that were identified as *C. lupus* ssp. (A) Frontside of an uncoloured fur scrap, (B) backside of the same uncoloured fur scrap, (C) frontside of a dyed fur scrap, (D) entire dyed fur. Photo credit: Luisa Garofalo (A, B, C) and Alessia Mariacher (D).

A total of 25 casework samples consisting of furs, pelts and scraps (Fig. 1) were submitted to molecular analyses to test the protocol. Samples were withheld at customs by the CITES (Convention on the International Trade in Endangered Species of Flora and Fauna) personnel, or seized during police searches in stores and at hawkers. Five furs were of old manufacturing, dating to mid of the last century, and six were artificially dyed. The species of origin was declared only for 10 items.

### Primer design

Dog and coyote are closely related canids that are both used in fur industry. In order to identify a sequence able to distinguish them, the complete mtDNAs of five dogs and four coyotes available from GenBank (http://www.ncbi.nlm.nih.gov/genbank/index.html) were downloaded and aligned (accession numbers in Table S1). A primer pair (furND1, Table 1) was designed on conserved flanking blocks of a 187 base pair (bp) long segment in the NADH ubiquinone oxidoreductase subunit ND1 coding gene, the most informative sequence in the alignment. The segment showed 12 nucleotide sites differing between dog and coyote. In order to use the homologous ND1 regions as a diagnostic marker to identify all our reference canids and felids, and possibly the other non-canid/felid species, we verified in silico, through the alignment of online sequences (Table S1), that interspecific variability was adequate to distinguish them unambiguously. Currently, ND1 gene is commonly used as genetic marker in mammals, and more than 58,000 sequences were deposited in the GenBank repository at the time of writing. Finally, we tested in vitro for positive ND1 amplifications and sequencing. Since the alignment for primer design was based on few sequences per species, intraspecific variability was not actually considered.

**Table 1:** Details on primer pairs for species identification in fur samples.

Primer set	Target mtDNA	Forward (5′-3′)	Reverse (5′-3′)	Amplicon size (bp)	Primer positions	*T*_a_ (°C)	Needs sequencing
furND1	ND1	CTAGCAATTATTCTCCTATCAGTCC	ACTAGTTCGGATTCTCCTTCAGT	187	3,183–3,370 U96639^1^	53	YES
lupCR	CR	GTGCTATGTCAGTATCTCCAGGT	GAAAGATCAGTGAATTATGAGATTGAG	149	15,494–15,643 U96639^1^	56	NO
nycCR	CR	ATACCTCACCACCCTCTATTT	TGATTAAGTAGTTTATGTCTATTGTGAC	155	15,468–15,623 NC013700^2^	52	NO
vulCR	CR	ATTAACCAGTGTGTTACGACTATCC	TGCAGTCATGTATGCTCCTGATA	169	15,469–15,638 AM181037^3^	56	NO
proCR	CR	CCATCGCATACTCAATCCTACTG	TCTGCGACGTAGGCCTATTTACTA	173	1,574–1,747 AB297804^4^	53	NO
felCytb	cytb	CCTAGTAGCGGATCTCCTAACC	ACGGTTTTCAATAATGCCTGAG	145	16,019–16,164 U20753^5^	55	YES

**Notes:**

Amplicon sizes include primer lengths. bp, base pair; *T*_a_, annealing temperature; ND1, NADH ubiquinone oxidoreductase subunit ND1 gene; CR, control region, cytb, cytochrome b. Primer positions according to reference complete mitochondrial sequences.

1 Kim et al. (1998);

2 published only in database;

3 Arnason et al. (2006);

4 published only in database;

5 Lopez, Cevario & O’Brien (1996).

In our workflow, amplification and sequencing with furND1 non-specific primer pair is performed as a first test to identify the species; afterwards a confirmation test should follow. To confirm the ND1 results with such an additional test, we selected four mitochondrial segments in the highly variable CR that were specific to dog, raccoon dog, fox and raccoon, respectively. Based on the online available entries, we aligned three or more sequences per species (Table S1) and designed four primer pairs (lupCR, nycCR, vulCR, proCR; Table 1) on nucleotide sites showing interspecific variation to obtain species-specific amplification products of 149, 155, 169 and 173 bp in length, respectively. Successful amplifications were verified in vitro for each target species. In felids, noncoding CR includes repeated interspersed sequences (Lopez, Cevario & O’Brien, 1996) that can complicate setting up a CR-based diagnostic test for species authentication. Therefore, in order to develop a confirmation test to identify cat furs after the ND1-based results, we selected a variable sequence in the cytb gene. Since we could not identify binding sites on the aligned sequences that were specific to *F. silvestris* DNA (i.e. different *Felis* and non-*Felis* species could amplify), alternatively we designed a non-specific primer pair (felCytb, Table 1) targeting a 145 bp long segment differing by nine nucleotides between domestic cat and Eurasian lynx (*Lynx lynx* was our reference felid species that was most closely related to *F. silvestris*). *Canis* and *Homo* were intentionally excluded from binding. Amplicons from felCytb PCRs were directly sequenced to eventually identify the species. To obtain successful amplifications in fur samples with degraded and poor DNA quality, all primers were designed to yield PCR products less than 200 bp in size. Homologous sequences from *H. sapiens* were included in all alignments to exclude human DNA as target for primer binding. PCR primer design followed the recommendations for animal DNA forensics as outlined in Linacre et al. (2011) and Linacre & Tobe (2013). The Multiple Alignment program included in the package Vector NTI v9.1 (Invitrogen, Carlsbad, CA, USA) was used to align sequences under the default settings. The software Primer3 (http://bioinfo.ut.ee/primer3-0.4.0/) was used for primer design.

### DNA extraction

DNA of the reference samples was isolated from approximately 25 mg of muscle using the QIAamp DNA Mini Kit (Qiagen, Hilden, Germany). DNA of the questioned samples was recovered from approximately 1 cm^2^ of fur product, consisting of both pelt and hair roots, if any. The extraction protocol followed the QIAamp DNA Mini Kit instructions, except for a slight modification in the lysis step, that was carried out in a Thermomixer® Comfort (thermoblock for 2 ml tubes; Eppendorf, Hamburg, Germany) at 56 °C for 18–24 h in 540 μl ATL buffer (Qiagen, Hilden, Germany), 60 μl proteinase K (20 mg/ml) and 40 μl 1 M DTT. The DNA was eluted into 100 μl AE buffer (Qiagen, Hilden, Germany). Three independent extractions were performed for each fur, to increase the chance for at least one positive amplification. DNAs from the reference and questioned samples were extracted in separated rooms and in different days to avoid cross contamination between high-DNA-quantity and low-copy-number (LCN) DNA samples. Two mock tubes with reagents and no DNA were included in each extraction session. Quantification of DNAs from the reference samples was obtained with the QuantiFlor® dsDNA system (Promega, Madison, WI, USA), whereas the amount of DNA isolated from furs was not measured. Quantification of whole DNA in fur/pelt samples, containing LCN DNA and, presumably, high levels of contamination from human DNA, will overestimate the number of target mitochondrial genomes (cf. Tobe & Linacre, 2008), thus making quantification unreliable.

### Amplification and sequencing

Each PCR reaction contained 2.5 μl of 10× buffer Gold (Applied Biosystems, Foster City, CA, USA), 200 μM each dNTP, 1.5 μM each primer, 2.5 mM MgCl_2_, 2.5 ng BSA, 1 U of AmpliTaq Gold Polymerase (Applied Biosystems, Foster City, CA, USA) and approximately 50 ng of DNA (or 5 μl of eluted DNA from questioned samples), in 25 μl total volume. Thermal cycling was performed in a Veriti system (Applied Biosystems, Foster City, CA, USA), and consisted of an initial 5 min denaturation step at 95 °C, 35 cycles of 30 s at 95 °C, 30 s at the annealing temperature (Table 1), 2 min extension step at 72 °C, followed by 5 min at 72 °C. Conditions for amplification of questioned samples differed from those of reference samples only in the number of cycles, that were increased to 40.

While furND1 and felCytb assays require sequencing of the amplified products, species-specific singleplex PCRs (lupCR, nycCR, vulCR, proCR) involve visualisation of bands under UV light following electrophoresis on 2% agarose gel and staining with Gel Red™ (Biotium, Inc., Hayward, CA, USA). A 50–2,000 bp DNA ladder (Sigma-Aldrich Chemicals, Milan, Italy) was used to verify the molecular sizes of amplicons. For the purposes of test validation, target amplicons from specific PCRs were sequenced to verify species identity, even though no sequencing will be needed in the routine. PCR products were cleaned up with the QIAquick PCR purification kit (Qiagen, Hilden, Germany) and sequenced bidirectionally using the amplification primers and the BigDye Terminator kit v3.1 (Applied Biosystems, Foster City, CA, USA) according to the manufacturer’s protocol. Unincorporated dyes and other contaminants were removed with the Agencourt® CleanSEQ solution (Beckman Coulter, Beverly, MA, USA), then sequences were loaded onto a 3130 Genetic Analyzer (Applied Biosystems, Foster City, CA, USA), and analysed using the Sequencing Analysis Software v5.3.1 (Applied Biosystems, Foster City, CA, USA). The ContigExpress program in Vector NTI v9.1 was used to visualise, edit the resulting sequences, and to obtain contigs from forward and reverse sequences. Questioned samples were processed using dedicated reagents, pipettes and thermal cycler. Prior to their use, pipettes and all disposable equipment were placed under a UV hood for decontamination. Amplifications of the three extracts from each fur sample were checked on the gel, and only the most intense product was purified and sequenced. Two extraction controls and two PCR negative controls were included in each amplification round. No positive controls were used when amplifying questioned samples to prevent any contamination.

### Validation studies

All specimens of the reference species were used to validate both species-specific and non-specific primer pairs to ensure that cross reaction with non-target DNA did not occur, and that human contamination was not detected. Sensitivity and repeatability of all assays were tested (using one single individual per reference species) through three independent replicates of a 10-fold DNA serial dilutions (from nearly 100 ng to 0.010 pg) to determine the lowest template limit of detection (LOD) and variation in the performance of different replicates (Dawnay et al., 2007). In order to assess the reproducibility of results, the tests were repeated by three operators. For species-specific primers, amplification products of the expected size were sequenced and submitted as queries to the BLAST algorithm (https://blast.ncbi.nlm.nih.gov/Blast.cgi) to check species identity. Sequenced amplicons from questioned samples were compared with both online and in-house reference sequences. Artificially mixed samples of the target species with human DNA (ratios: 1:1, 1:10, 1:100) were analysed to verify the ability of primers to detect the target species at the determined values of LOD (see Results) without being affected by human contamination. Mixtures were tested with species-specific primers using the relevant target DNA. Non species-specific primers were tested using separately dog and cat DNAs with furND1 pair, and cat DNA with felCytb pair. Amplicons from 1:100 mixtures (i.e. the ones with the highest amount of human DNA) were sequenced to verify that only the target DNA was amplified. To reach a conclusive identification of species, we arbitrarily used a threshold of 98% for the sequence match between questioned samples and the entries held on public databases or our in-house reference sequences (see Lee et al., 2009; Linacre & Tobe, 2013). Validation tests were undertaken under the recommendations of the Scientific Working Group for Wildlife Forensic Sciences (SWGWILD, 2013) and Animal, Plant and Soil Traces (http://enfsi.eu/about-enfsi/structure/working-groups/animal-plant-and-soil-traces/) expert working group inside the European Network of Forensic Science Institutes (ENFSI, 2016).

## Results

### DNA testing on reference samples

DNA from all specimens of the reference species were tested with primers furND1. Amplification reactions yielded products of the expected size in all canid and felid samples (Table 2). Positive PCRs were also obtained for *M. foina*, *L. lutra* and *S. vulgaris*, while multiple bands of non-expected sizes amplified in *O. cuniculus*. No amplification occurred in *P. lotor*, *L. europaeus* and *H. sapiens*. Amplicons were sequenced bidirectionally (Raw data S1), and sequences were submitted to the GenBank database for similarity search to verify their identity. Dog and coyote sequences showed 100% similarity with the corresponding online entries. At the time of primer design, homologous sequences from *C. aureus* were not lodged in GenBank and could not be compared with our results. However, our jackal sequence differed by seven and 16 diagnostic sites from those of dog and coyote, respectively. Later, three complete mitochondrial sequences were published online. Comparisons showed that our ND1 segment matched that of the Eurasian jackal, and the diagnostic sites were confirmed (Table S1).

**Table 2:** Presence (YES) or absence (NO) of amplification products in the reference species using non-specific and specific primer sets.

Family	Species	Primer set					
		furND1	lupCR	nycCR	vulCR	proCR	felCytb
Canidae	*Canis lupus* ssp. (dog/wolf)	YES (0.091)	YES (0.103)	NO	NO	NO	NO
Canidae	*Canis latrans* (coyote)	YES (0.011)	NO	NO	NO	NO	NO
Canidae	*Canis aureus* (jackal)	YES (0.120)	NO	NO	NO	NO	NO
Canidae	*Nyctereutes procyonoides* (raccoon dog)	YES (0.048)	NO	YES (0.025)	NO	NO	NO
Canidae	*Vulpes vulpes* (red fox)	YES (0.105)	NO	NO	YES (0.110)	NO	NO
Felidae	*Felis silvestris* ssp. (domestic/wild cat)	YES (0.045)	NO	NO	NO	NO	YES (0.108)
Felidae	*Lynx lynx* (Eurasian lynx)	YES (50)	NO	NO	NO	NO	YES (49.8)
Felidae	*Panthera tigris* (tiger)	YES (2)	NO	NO	NO	NO	YES (19.8)
Procyonidae	*Procyon lotor* (raccoon)	NO	NO	NO	NO	YES (1.1)	NO
Mustelidae	*Martes foina* (beech marten)	YES	NO	NO	NO	NO	NO
Mustelidae	*Lutra lutra* (otter)	YES	NO	NO	NO	NO	NO
Leporidae	*Oryctolagus cuniculus* (rabbit)	Aspecific bands	NO	NO	NO	NO	NO
Leporidae	*Lepus europaeus* (brown hare)	NO	NO	NO	NO	NO	NO
Sciuridae	*Sciurus vulgaris* (red squirrel)	YES	NO	NO	NO	NO	NO
Hominidae	*Homo sapiens* (human)	NO	NO	NO	NO	NO	NO

**Note:**

Values of LOD (limit of detection) DNA in picograms (pg) are in parentheses. LODs were obtained only in canid and felid species using furND1 primer pair and in the target species using the other primer sets.

Between 98% and 100% match with online sources was found for raccoon dog, fox, cat, lynx, tiger, beech marten, otter and red squirrel. Tests for sensitivity of furND1 primers were conducted only for canid and felid species, using one individual per species (as it was adopted in all subsequent validation tests). Values of LOD (Table 2) ranged from 0.011 pg in *C. latrans* to 50 pg in *L. lynx.* Results from three replicates yielded 100% repeatability of the tests. Same results were obtained when analyses were conducted by three operators, thus yielding 100% reproducibility. Mixed samples were artificially prepared separately with dog and cat DNA at their LODs (0.091 and 0.045 pg, respectively) plus human DNA at increasing amounts according to the ratios 1:1, 1:10 and 1:100. They all yielded positive amplifications. Sequencing of PCR products from 1:100 mixtures allowed correct species identifications in either dog/human or cat/human mixtures.

Species-specific primers were designed either to support the results from the furND1 test, as in the case of *C. lupus* ssp., *V. vulpes* and *N. procyonoides*, or to definitively diagnose the species via positive amplification, as in the case of *P. lotor*, that did not amplify with the furND1 primers (Table 2). The primer pair felCytb was used to confirm felid species via sequencing. Primer sets were tested with target and non-target reference samples to verify their specificity. Amplifications using lupCR, nycCR, vulCR and proCR primers produced bands of the expected size in the target species, while no products were amplified in non-target species (Table 2). Once entered in the BLAST algorithm, all sequenced amplicons matched the correct species with 99–100% homology. Values of LOD for specific bands ranged from 0.025 pg for nycCR primer pairs to 1.1 pg for proCR. Primers felCytb yielded a band of the expected size in *F. catus*, *L. lynx* and *P. tigris*, while no bands were observed in non-felid samples. LODs for felCytb ranged from 0.108 pg in *F. catus* to 19.8 pg in *P. tigris* and 49.8 pg in *L. lynx*, with a binding efficiency of primers that was approximately 20- and 50-fold higher for cat DNA than for tiger and lynx DNA, respectively. Lower affinity of felCytb primers for non *Felis* sequences was likely due to mismatches (point mutations) occurring at the binding sites. Same LODs were obtained from three PCR replicates (100% repeatability) for all specific and non-specific primer pairs. Three sets of PCR replicates were carried out by different operators, and 100% reproducibility was obtained. Bands from specific assays (at the determined LODs) produced sequences that correctly identified the target species with 99–100% similarity when compared to the sequences in the online and in-house databases. Mixed samples tested with both species-specific (lupCR, nycCR, vulCR, proCR) and non species-specific (felCytb) primers yielded positive amplifications up to concentrations of human DNA that were a hundred times higher than their LODs. Sequencing of PCR products from mixtures all yielded correct identifications of species.

### Questioned samples

DNA amplifications produced positive results in 21 fur samples (Table 3). Of these, some (FUR-10, -17, -18, -19) were very old, dating back to mid of last century, while others (FUR-7, -12, -22, -23) were dyed, suggesting that age and chemical staining do not necessarily affect positive amplifications. Despite multiple DNA extractions and amplifications with all the available primer sets, no results were ever obtained from furs FUR-3, -5, -15 and -25, presumably either because the DNAs were too degraded to yield any PCR product or due to the absence of binding sites for primers in non-target species (e.g. *L. europaeus* and *O. cuniculus*, see Table 2). The use of both identification (furND1) and confirmation (lupCR, nycCR, vulCR, proCR, felCytb) primer sets allowed us to assign the species to 18 fur samples. Two felid furs (FUR-19, -20) could only be identified at the genus level, *Felis* and *Lynx*, respectively. Less than 98% sequence homology was obtained between sequences from these fur samples and either our reference species or any sequence (both ND1 and cytb) on the online sources. The low percentage matches were best explained by the lack of our query species in the public databases, rather than by the presence of higher than expected intraspecific variation. Among the non-canid and non-felid species, the use of furND1 non-specific primer pair identified *Neovison vison* (FUR-16) and *Sciurus* spp. (FUR-9). Primer pair vulCR was confirmed to anneal exclusively to the red fox *V. vulpes* DNA (FUR-10, -11, -13, -14). In fact, the phylogenetically close arctic fox *Alopex lagopus* DNA (FUR-12, -24) did not yield any vulCR amplification product. Five out of 21 samples belonged to *C. lupus* ssp. (Fig. 1). Among 10 furs for which the source species were declared, seven were confirmed (one fur yielded no DNA). Conversely, two samples, declared as wolf (FUR-18) and siberian wolf (FUR-17) furs were rather coyote *C. latrans*.

**Table 3:** Results from caseworks.

ID	Approximative age	Dyed	Declared AS	DNA	Primer set(s)
FUR-1	1980–2016	No	raccoon dog	*Nyctereutes procyonoides* (raccoon dog)	furND1 + nycCR
FUR-2	1980–2016	No	raccoon dog	*Nyctereutes procyonoides* (raccoon dog)	furND1 + nycCR
FUR-3	1980–2016	No	raccoon dog	No amplification	All
FUR-4	1980–2016	No	nd	*Nyctereutes procyonoides* (raccoon dog)	furND1 + nycCR
FUR-5	1980–2016	No	nd	No amplification	All
FUR-6	1980–2016	No	nd	*Canis lupus* ssp. (dog/wolf)	furND1 + lupCR
FUR-7	1980–2016	Yes	nd	*Canis lupus* ssp. (dog/wolf)	furND1 + lupCR
FUR-8	1980–2016	No	coyote	*Canis latrans (coyote)*	furND1
FUR-9	1980–2016	No	squirrel	*Sciurus* spp. (squirrel)	furND1
FUR-10	1930–1980	No	fox	*Vulpes vulpes* (red fox)	furND1 + vulCR
FUR-11	1980–2016	No	silver fox	*Vulpes vulpes* (red fox)	furND1 + vulCR
FUR-12	1980–2016	Yes	nd	*Alopex lagopus* (arctic fox)	furND1
FUR-13	1980–2016	No	nd	*Vulpes vulpes* (red fox)	furND1 + vulCR
FUR-14	1980–2016	No	nd	*Vulpes vulpes* (red fox)	furND1 + vulCR
FUR-15	1980–2016	Yes	nd	No amplification	All
FUR-16	1980–2016	No	nd	*Neovison vison* (mink)	furND1
FUR-17	1930–1980	No	siberian wolf	*Canis latrans* (coyote)	furND1
FUR-18	1930–1980	No	wolf	*Canis latrans* (coyote)	furND1
FUR-19	1930–1980	No	wild cat	*Felis* spp. (domestic/wild cat)	furND1 + felCytb
FUR-20	1980–2016	No	nd	*Lynx* spp. (lynx)	furND1 + felCytb
FUR-21	1980–2016	No	nd	*Canis lupus* ssp. (dog/wolf)	furND1 + lupCR
FUR-22	1980–2016	Yes	nd	*Canis lupus* ssp. (dog/wolf)	furND1 + lupCR
FUR-23	1980–2016	Yes	nd	*Canis lupus* ssp. (dog/wolf)	furND1 + lupCR
FUR-24	1980–2016	No	nd	*Alopex lagopus* (arctic fox)	furND1
FUR-25	1930–1980	Yes	nd	No amplification	All

**Note:**

nd, not declared. See text for details.

## Discussion

The habit of wearing animal furs is decreasing in Western countries, and people are increasingly outraged and opposed to using pets in fur industry. In 2007, the EU acted as a spokesperson for these feelings and issued a Regulation that officially banned the use and import/export of dog and cat furs, hides or their derivatives in all Member States. However, the enforcement of legislation is difficult for many reasons, and the Regulation is actually disregarded in many EU countries, despite clear violations. As regards to laboratory testing, the obstacles are basically due to the use of degraded samples (furs and pelts), as well as to the difficulty of differentiating closely related species in canids and felids.

Mitochondrial markers were developed for amplification in degraded and poor quality DNA samples, and were validated on a reference collection of tissues obtaining very solid results in terms of specificity, sensitivity, repeatability and reproducibility. Moreover, sensitive and highly specific primer pairs proved effective in excluding human contamination from amplifications. The logic of our molecular steps is that a short segment in the mitochondrial ND1 gene is sequenced to provide a first identification of the species. Afterwards, a set of diagnostic species-specific markers is used to confirm the results. The amplification of two markers to eventually identify the species adds confidence and reliability to species diagnosis and is highly recommended in forensics (Linacre & Tobe, 2013). Additionally, the use of two markers enhances the chance for positive results in degraded samples: if one primer pair fails to bind for some reasons, a second chance of successful amplification with the second pair will still stand.

In caseworks, we identified *C. lupus* ssp. in five samples, that were probably illicit furs, while the felid fur was identified only at the genus level *Felis* spp., so that its (il)legality could not be ascertained. Two samples that were declared as originating from wolf and siberian wolf, respectively, were actually both coyote furs. This suggests that commercial frauds are commonly committed through deliberate mislabelling and selling of *aliud pro alio*.

The present molecular procedure turned out to be a good starting point for enforcing the EU Regulation against dog and cat fur trade. However, we are fully aware that both specificity and sensitivity of the method can be improved. DNA-based distinction between wolf and dog, for example, can be made relatively easily at the local level (Randi & Lucchini, 2002; Godinho et al., 2011; Lorenzini et al., 2014), but it represents a major challenge on a global geographic scale, when the source wolf population is unknown or when there are no population databases available. Dog domestication dates back to 135,000–15,000 years ago (vonHoldt et al., 2010; Larson & Bradley, 2014; Skoglund et al., 2015): despite a wide range of estimates, this remains a short evolutionary time frame indeed, and the two taxa are phylogenetically very closely related. Additionally, the wolf is legally hunted and potentially used as fur-bearing animal in some EU Member States, as well as in most areas of its distribution worldwide. The same holds true for the domestic cat and its wild *F. silvestris* counterparts, that are hardly distinguishable both phenotypically and genetically (O’Brien et al., 2008; Oliveira et al., 2015). To face these issues at the molecular level, we are developing more sensitive and specific tests via real time PCR amplification of both mtDNA markers and published nuclear Single Nucleotide Polymorphisms (vonHoldt et al., 2010; Kurushima et al., 2013). The ultimate aim is to discriminate efficiently closely related taxa in highly degraded samples, like furs and fur products, in forensic contexts where source attribution is essential to the assignment of responsibilities.

## Conclusion

For over 10 years now, the EU has issued a Regulation that prohibits the use and trade of dog and cat furs, but violations of law are recurrent in all Member States. One of the reasons for that is the lack of an official, validated analytical protocol to exclude these species as source of furs. The Regulation provides only vague indications on the methods to use, and data on their respective effectiveness in detecting dog and cat furs are completely lacking. Among the methods suggested, we have explored a molecular DNA approach as a best compromise between feasibility (DNA laboratory with basic facilities and good laboratory practices) and diagnostic power (closely related species can be reliably distinguished). It is nonetheless true that ample room for improvement is still possible for this method, and we are working in this direction. The use of our non-specific primer pairs (furND1 and felCytb) can also be extended to the identification of wild species that are victims of poaching and illegal harvest for trade, such as the endangered, protected and CITES-listed species.

To sum up, major outcomes and advantages of our DNA-based protocol are:

1. sensitive and informative molecular marker for initial first identification of species;
2. species-specific markers to confirm the results through an inexpensive end-point PCR or by sequencing;
3. short PCR amplicons for the analysis of degraded and poor quality DNA sample;
4. binding primers that avoid contamination and interference from human DNA;
5. user-friendly protocol for any laboratory equipped for analysis of LCN DNA.

## Supplemental Information

10.7717/peerj.4902/supp-1 Supplemental Information 1 Sequences of our reference specimens for ND1 and cytb markers. Sequences were not submitted to GenBank because they are shorter than the minimum length requested for submission (200 base pairs). Due to lack of intraspecific variability of the analysed sequences, one sequence per species is shown.

10.7717/peerj.4902/supp-2 Supplemental Information 2 Accession numbers of the sequences used to design primers.

## Additional Information and Declarations

### Data Availability

The following information was supplied regarding data availability: The sequences are uploaded as raw data in a Supplemental File. Sequences were not submitted to GenBank because they are shorter than the minimum length requested for submission (200 base pairs). Due to lack of intraspecific variability of the analysed sequences, one sequence per species is shown.
