# Supplementary material for: Hindering the illegal trade in dog and cat furs through a DNA-based protocol for species identification
Source: PeerJ. 2018 Jun 5;6:e4902. doi: 10.7717/peerj.4902 (PMC5993017; doi:10.7717/peerj.4902)
Supplement: Supplemental Information 1 — Sequences were not submitted to GenBank because they are shorter than the minimum length requested for submission (200 base pairs). Due to lack of intraspecific variability of the analysed sequences, one sequence per species is shown. [file peerj-06-4902-s001.pdf]

Raw data S1. Sequences of our reference specimens for ND1 and cytb markers. Sequences were not submitted to GenBank because they are shorter than the minimum length requested for submission (200 base pairs). Due to lack of intraspecific variability of the analysed sequences, one sequence per species is shown.

ND1 reference sequences:

*Canis lupus*_ND1
TCCTAATAAACGGGTCATTTACACTATCCACGCTAATTATTACCCAAGAACATATATGA
TTAATCTTTCCGGCCTGACCCCTAGCCATGATATGATTCATCTCTACCCTAGCAGAAAC
TAATCGAGCCCCCTTCGACTTA

*Canis latrans*_ND1
TTCTAATAAATGGGTCATTCACATTATCTACGCTAATCATTACCCAAGAGCATATATGA
TTAATCTTTCCAGCTTGACCCCTAGCCATGATATGATTCATTTCCACTCTAGCAGAGAC
TAATCGAGCCCCCTTCGACTTA

*Canis aureus*_ND1
TCCTAATAAACGGGTCATTTACACTATCCACACTAATTATCACCCAAGAACATATATGA
CTAATCTTTCCGGCCTGGCCCCTAGCCATGATATGATTCATTTCTACTCTAGCAGAAAC
TAACCGAGCCCCCTTCGACTTA

*Nyctereutes procyonoides*_ND1
TACTGATAAACGGATCCTTCACATTATCCACACTCATTATCACCCAAGAACACATATGA
TTAATTTTTCCAGCCTGACCATTAGCCATAATATGATTTATTTCCACACTAGCAGAAAC
AAACCGAGCCCCCTTCGACCTA

*Vulpes vulpes*_ND1
TGCTAATAAACGGATCATTCACACTATCCACACTCATTATTACCCAAGAGCATATATGA
CTAATTTTCCCTGCCTGACCTCTAGCCATGATATGATTTATCTCTACCCTAGCAGAAAC
AAACCGGGCCCCCTTTGACTTA

*Felis silvestris*_ND1
TACTAATAAACGGATCCTTCACACTAGCCATACTAATCACCACTCAAGAATATGTGTGA
CTAATCATTCCTGCATGACCCCTAGCCATAATATGATTTATCTCAACCCTAGCAGAGAC
CAATCGAGCCCCATTCGACCTG

*Lynx lynx*_ND1
TACTAATAAATGGATCCTTCACACTAGCCACACTAATCACCACCCAAGAATACATGTG
ACTAATTATCCCTGCATGACCCCTAGCCATAATATGATTCATCTCAACACTAGCAGAAA
CCAACCGAGCCCCATTCGACCTA

*Panthera tigris*_ND1
TACTAATAAATGGATCCTTCACATTAGCTGCACTAATTACCACCCAAGAATACATCTGG
CTCATCATCCCTGCATGACCCCTAGCCATAATATGATTCATCTCCACACTAGCAGAAAC
CAACCGAGCTCCATTTGATCTA

*Martes foina*_ND1
TATTAATAAATGGCTCTTTTACCCTATCCACACTTACTACTACACAAGAACACCTATGA
CTAATCCTCCCCACATGACCCCTAGCTATAGTATGATTTATCTCAACCCTAGCAGAAAC
CAACCGCACCCCATTTGACTTA

*Lutra lutra*_ND1
TACTAATAAACGGCTCATTCACCCTATCCACATTAATCACCACACAGGAGCACCTATGA
CTAATCCTACCCACATGACCCCTGGCTATAATATGATTTATTTCAACTCTGGCAGAAAC
TAATCGCGCACCATTTGATCTA

*Sciurus vulgaris*_ND1
TCCTTATAAACGGCTCATTCACCCTATCAACCCTTATCACAACCCAACAATTCATATGA
CTAATTTTACCAACCTGACCCTTGGCTATAATATGATTTATTTCTACCCTAGCTGAAAC
AAATCGAGCCCCATTTGACCTA

Cytb reference sequences:

*Felis silvestris*_Cytb
CTAACATGAATCGGTGGCCAACCTGTAGAACATCCATTCATCACCATCGGCCAACTAG
CCTCCATCCTATATTTCTCAACCCTCCTAATCCTAATACCCAT

*Lynx lynx*_Cytb
CTAACATGAATTGGCGGCCAACCCGTAGAACACCCTTTCATCACCATCGGCCAACTGG
CCTCCATCCTATACTTCTCAAC TCTCCTAGTCCTAATACCCAT

*Panthera tigris*_Cytb
CTAACATGAATTGGTGGCCAACCTGTAGAACACCCCTTTATTGCCATCGGCCAACTAGC
CTCTATCCTATACTTCTTCATCCTCCTAGTCTTAATCCCCAT
