# Supplementary material for: Hindering the illegal trade in dog and cat furs through a DNA-based protocol for species identification
Source: PeerJ. 2018 Jun 5;6:e4902. doi: 10.7717/peerj.4902 (PMC5993017; doi:10.7717/peerj.4902)
Supplement: Supplemental Information 2 [file peerj-06-4902-s002.pdf]

Table S1. Accession numbers of the sequences used to design primers.

SPECIES	COMPLETE mtDNA	ND1	CONTROL REGION	CYTB
Canis lupus<br>dog/wolf	U96639<br>EU442884<br>FJ032363<br>AM711902<br>DQ480505	U96639<br>EU442884<br>FJ032363<br>AM711902<br>DQ480505<br>AH014072	U96639<br>EU442884<br>FJ032363<br>AM711902<br>DQ480505	U96639<br>EU442884<br>FJ032363<br>AM711902<br>DQ480505
Canis latrans<br>coyote	DQ480509<br>DQ480510<br>DQ480511<br>EU789789	DQ480509<br>DQ480510<br>DQ480511<br>EU789789	DQ480509<br>DQ480510<br>DQ480511<br>EU789789	DQ480509<br>DQ480510<br>DQ480511<br>EU789789
Canis aureus<br>jackal		KT448272<br>KT448273<br>KT448274	AY289996<br>AY289997<br>AF184048	
Nyctereutes procyonoides<br>raccoon dog		NC013700<br>GU256221<br>KF709435	FJ888513<br>FJ888514<br>NC013700<br>GU256221	
Vulpes vulpes<br>red fox		JN711443<br>GQ374180<br>AM181037<br>KP342452	JN711443<br>GQ374180<br>AM181037	JN711443<br>AM181037
Procyon lotor<br>raccoon		AB297804<br>AB462045<br>AB462046<br>AB462049	AB297804<br>AB361247<br>AB462046<br>AB462047<br>AB462048<br>AB462049	
Felis silvestris<br>domestic/wild cat		U20753		U20753<br>AB194812<br>AB004237<br>AB004238<br>AB194813<br>AB194814<br>AB194815<br>AB194816<br>AB194817<br>X82296<br>AY170102<br>EF689046
Lynx lynx<br>Eurasian lynx		KM982549		KM000081<br>KM000082<br>KF990332
Panthera tigris<br>tiger		EF551003<br>KF892541<br>KJ508412<br>KJ508413		AF053024<br>EF551003<br>KC495059<br>KC879293<br>KF892541<br>KJ508412<br>KJ508413
Homo sapiens<br>human		HM357817<br>KF384026<br>KJ154733<br>KC345898<br>JX900490<br>JN655842	HM357817<br>KF384026<br>KJ154733<br>KC345898<br>JX900490<br>JN655842	HM357817<br>KF384026<br>KJ154733<br>KC345898<br>JX900490<br>JN655842
